# Contesting restrictive mobility norms among female mentors implementing a sport based programme for young girls in a Mumbai slum

**DOI:** 10.1186/s12889-018-5347-3

**Published:** 2018-04-10

**Authors:** Shweta Bankar, Martine Collumbien, Madhumita Das, Ravi K. Verma, Beniamino Cislaghi, Lori Heise

**Affiliations:** 1 International Centre for Research on Women, 603, The Affaires, Sector 17, Sanpada, Palm Beach Road, Navi Mumbai, Mumbai 400705 India; 2 London School of Hygiene and Tropical Medicine, London, UK; 3 CREA, New Delhi, India; 4 International Centre for Research on Women, Delhi, India; 5 Johns Hopkins Bloomberg School of Public Health and School of Nursing, Baltimore, MD USA

**Keywords:** Gender norms, Mobility, Collective agency, Sports-based programme, India

## Abstract

**Background:**

Harmful gender norms are known structural barriers to many public health and development interventions involving adolescent girls. In India, restrictions on girls’ liberty to move freely in public spaces contribute to school dropout and early marriage, and negatively affect girls’ health and wellbeing, from adolescence into adulthood. We report on mechanisms of change among female mentors 18 to 24 years old who contested discriminatory norms while implementing a sports-based programme for adolescent girls in a Mumbai slum.

**Methods:**

We adopted a prospective qualitative research design. Our analysis is based on case studies derived from two rounds of face to face, in -depth interviews with 10 young women recruited to serve as mentors for the project’s young female athletes. We combined both thematic and narrative analysis.

**Results:**

The programme created opportunities for collective action, increasing mentors’ ability to think and relate in a collectivized manner, and challenged the traditional female identity constructed for young women, which centres on domestic duties. The mentors themselves negotiated freedoms both in and outside their homes, which required careful and strategic bargaining. They changed the nature of key day-to-day social interactions with parents and brothers, as well as with neighbours, parents of their groups of athletes and men on the streets. They formed a new reference group for each other in terms of what was possible and acceptable. Demonstrating greater negotiation skills within the family helped win parents’ trust in the mentor’s ability to be safe in public spaces. Parents became active supporters by not giving into social sanctions of neighbours and relatives thus co-producing a new identity for their daughters as respectable young women doing ‘good work’. They effectively side stepped reputational risk with their presence in public spaces becoming de-sexualised.

**Conclusions:**

Mentors contested mobility restrictions by taking risks as a group first, with collective agency an important step towards greater individual agency. This research provides important insights into addressing embedded social norms that perpetuate gender discriminatory practices and the social patterning of health inequalities.

## Background

Addressing gender inequality is recognized as essential to health and sustainable development. Gender inequality and early marriage are obstacles to women’s life chances as they impact negatively on education, and lead to early child-bearing and increased risk of maternal and child morbidity and mortality [1]. Adverse consequences persist across the woman’s life-course and into the next generation, perpetuating cycles of poverty and ill-health among the poorest. In India, marriage before age 18 is legally banned. Widespread efforts by both government and non-government organisations to curtail early marriage are helped by strong economic growth and increased education. Indeed, the latest National Family Health Survey in 2015 reports significant progress in delaying marriage and improving levels of education. In urban Maharashtra, the proportion of women age 20–24 years who married before age 18 dropped from 29 to 18% in 10 years. The overall proportion of women with 10+ years of education increased from 31% to 42, and is at 52% in urban areas [2]. For marginalised slum communities, conditions are less favourable with other structural inequalities compounding gender inequality.

Gender norms are powerful pervasive beliefs about gender-based social roles and practices that are deeply embedded in social structures. Norms are produced by patriarchal power-relations and maintained, in part by self-surveillance as girls follow internalised gender scripts. In India, gender norms produce outcomes that disadvantage girls and women. Norms reinforce women’s caregiving and reproductive roles and limit their access to public space. Gender-related scripts were found more significant than either economic rationales or women’s empowerment in explaining variations in age at marriage across India [3]. While empowerment theories have explored women’s subordination within the household, social institutions, and the state, they have paid insufficient attention to the symbolic aspects of gender [3]. According to the “performance theory” of gender, girls learn to perform gender roles and adopt gender identities that ‘regulate’ social interactions and limit options including the freedom to move and be visible in public space [4–6]. It shapes their choices in ways that continuously reproduce gendered pattern of behaviour in day-to-day social interactions. It is the visible display of gender that allows neighbours and communities to police women’s actions. The anticipation of social sanctions or rewards, with the need for belonging and approval from the group, is one of the key motivators for compliance [7].

Once a girl reaches menarche in India, parents get concerned about demonstrating her ‘good’ virtue, an essential aspect for finding a good matrimonial match [3]. Good virtue requires modesty, a deferential demeanour, proficiency in household chores and above all sexual purity. Early marriage becomes part and parcel of the symbolic display of segregation, modesty, and chastity. The supremacy of virginity means unmarried girls are in need of both restraint and protection, limiting their access to public spaces [3]. Gendered power dynamics are embedded in space, organised into the “masculine” public domain, and the “feminine” private sphere [8]. Popular discourse associates women’s safety with the modesty of her clothing, with *burkhas* and *salwar kameez* designed to hide the female body from public view. As custodians of family honour, girls are socialised to fear not only potential violence in public spaces but also the threat of public censure that will impact her ‘reputation’ [5]. The fear of sexual harassment maintains male privilege, diminishes women’s feelings of safety and belonging in public places and restricts their freedom of movement [9]. Fear and social control significantly limit girls’ individual agency to access public space, a structural barrier in any intervention aiming to increase female education and participation as citizens in society.

Any emancipatory change among adolescent girls thus requires concurrent social change. Gender constructions can be challenged and redefined when performance of gendered identity transgresses established norms [10]. Where individual agency is largely constrained by structural factors, social change starts at the level of social practices: via changes in day-to-day social relations that individuals and communities cultivate [11]. There is very little detailed description and analyses of the subtle changes that occur when women contest discriminatory norms in gender programmes and this paper addresses this gap using research data collected in the context of an intervention that encourages the inclusion of young women and girls in public spaces.

### Parivartan

*Change at Play* is a sports-based programme among adolescent girls age 12–16, implemented in a slum community in Mumbai. Young women were recruited from within the slum community to serve as “mentors” to younger girls. They were trained to lead reflection sessions on gender and to coach *kabaddi*, a contact team sport. They then delivered a carefully structured programme of life skills and gender training to younger girls, interspersed with weekly games of *kabaddi*. Concepts from social norms theory [7, 12] were used to shape the content and implementation of the programme. To perform their responsibilities, mentors needed to challenge mobility restrictions and negotiate their visibility in public space. In this paper, we focus on the change process among mentors, noting how the programme enhanced their agency to strategize and negotiate greater freedom and visibility in their community. We emphasize changes in day-to-day social interactions at home and in public spaces.

## Methods

### Study setting and design

The Parivartan programme was located in four plots at the periphery of Shivaji Nagar, one of Mumbai’s largest urban slums. The community consists predominantly of Muslim migrant families mainly from Uttar Pradesh (northern India) working in the unorganized sector and living in substandard housing with poor water supply and sanitation. The wards included in this study are near a dumping ground and some lanes are seen as unsafe because drug users frequently loiter there. Various NGOs have been implementing a range of health and development projects including a sports-based programme for boys. Increasingly community development programmes work at both the individual and community level. Men in the communities are mobilised mainly via the *Imams* in the mosques and women via *Mahila mandals,* which are small groups of neighbours residing in the same lanes. Public spaces are dominated by men and after menarche, girls’ mobility and visibility are restricted, as is their interaction with boys. *Burkhas* or *salwar kameez* with headscarf are the requisite dress code outside the home.

Apnalaya, a grass-roots NGO with longstanding presence and credibility in the community, implemented the intervention. The research was implemented by the International Centre for Research on Women (ICRW), in collaboration with the STRIVE research consortium.

We adopted a prospective qualitative research design to study the interaction between the intervention and the changing context within which it was implemented [13]. Case studies were done with 10 mentors and 15 girls during the implementation phase, preceded by participatory research among girls, mentors, mothers and fathers at the design stage. Written consent to participate was obtained from all research participants.

### Sampling

Mentors for the intervention were recruited from within the slum, with eligibility based on age (18–24 years) and education (class 12+), as identified by Apnalaya (though educational qualifications were relaxed in some cases). Out of 40 applicants, 15 young women were selected as mentors after three rounds of personal interviews to establish their interest in sports, level of aspiration, their commitment towards the programme and their position on the rights of women and girls. They were coached on *kabaddi*, mentorship and leadership skills and trained in a Gender and Gender Equality curriculum during the initial 5-day residential programme. There were 4 more 2-day workshops (one of which was residential) on using the intervention tools with their group of athletes. All mentors were responsible for conducting two sessions per week (one reflective session and one sports session) with their group of female athletes. The full programme consisted of a well-structured curriculum encouraging reflection on gender expectations and myths, human rights and life skills delivered over 15 months. The tools designed for these sessions consisted of 21 Card Sessions presenting topics and 19 Group Education Sessions with role plays and games to facilitate practice and reflection on issues introduced the previous week. Mentors received a monthly stipend of INR 3000 (about 45 US $). Implementation challenges were discussed during weekly meetings with Apnalaya staff, who were available throughout the project to help mentors problem solve. Project staff placed special emphasis on helping mentors ensure their personal safety and that of their athletes.

### Data collection

By the time the implementation of programme started, five mentors had dropped out (the reasons for this attrition is discussed later), and the data used for this analysis draws exclusively on 2 rounds of face-to-face in-depth interviews with the 10 remaining mentors. The interview guides were developed based on literature on gender practices in India and understanding of the community, based on formative research. Guides for the second round were personalised with follow-up of particular issues of interest arising in the first round, while keeping style of interviewing and issues covered consistent. Interviews were shaped around a semi-structured interview guide with open-ended questions on: gains, achievements, difficulties and challenges encountered as Parivartan mentors; safety and security; commitment to and outcomes associated with their mentor roles; freedom to move around in the community and relaxation of restrictions; perceptions of changes in themselves and in the relationships with parents and caretakers; and changes in those whose opinion the mentor’s valued in terms of their own behaviour (their reference group).

All interviews were done by the first author at the ICRW field office, a comfortable and relaxing environment which enabled confidentiality, at a time convenient to them. Interviews were digitally recorded, transcribed and translated verbatim.

### Analysis

We combined thematic and narrative analyses [14]. The transcripts were reviewed and themes discussed continuously during data collection by the authors. We followed a step-wise procedure of familiarising ourselves with the data, identifying a thematic framework and developing a coding frame, using Atlas.ti (version 7.0.88) for data management. We also developed spreadsheets to explore commonalities and differences across mentors, while ensuring that the context and integrity of each narrative was maintained across both data rounds. We looked for patterns in how mentors negotiated space and exerted agency paying attention to the interactional context, acknowledging that narrative identities are shaped and co-constructed between mentor and interviewer [14]. Interpretation was influenced by insights gained during observations in the community and during regular meetings with mentors.

For this paper we investigate the theme of mobility, identifying how mentors negotiated the relaxation of mobility and visibility restrictions, both at home and in the community. We explore the extent to which the Parivartan programme gave them the skills and opportunities to do so, and how their personal history and circumstances may have impacted the trajectory of their transformation. We also examine the various strategies mentors developed as a group to contest norms and the factors contributing to break-downs and set-backs of achieved freedoms.

## Results

### Profile of mentors

Table 1 gives the profile of the 15 mentors who enrolled as mentors and started the residential training. This is a select sample of young women, well-educated compared to their contemporaries in the slum, with several pursuing a university degree. Mentor 1 and 5 were the only ones who had dropped out before finishing 12th class. Mobility restrictions had stopped mentor 4 from attending mainstream school after 10th class, but she studied from home and sat 12th class exams, though unfortunately failed. Mentors 10 and 14 studied at university level from home. Only mentor 11 was married at the time of recruitment, and lived with her in-laws. Most other mentors lived with both parents, except for mentor 2 whose parents had both died, and mentor 10 who lost her father. All families had previous contact with Apnalaya, some as active members of self-help groups, while others had been beneficiaries of schemes, including scholarships for children’s education. Mentors expressed different motives for becoming mentors – for some it was additional family income, for others it represented an opportunity to learn and come out of the house.

**Table 1:** Profile of mentors recruited into Parivartan programme

	Age	Profession/education at recruitment	Religion	Family composition	Family’s association with Apnalaya	Motivation to join Parivartan
M1	20	Not employed; Dropped out of school in class 10	Hindu	Middle child with 2 brothers. Lives with both parents.	Mother active member of various groups	Forming own identity
M2	19	Full-time student; First year of university	Muslim	First born with 1 brother and 1 sister. They live with grandmother after both parents died.	Mother employed by Apnalaya before her death - mentor is recipient of scholarship.	Additional income
M3	25	Nurse in hospital; Diploma in nursing	Muslim	Third-born with 2 brothers and 1 younger sister. Lives with both parents.	Mother active member; father participated in programs for men; all children volunteered/ participated.	Interest to work with girls in community
M4	19	Not employed; Appeared for class 12 exam (externally)	Muslim	Middle child with older brother and younger sister. Lives with both parents.	Father associated with rag pickers organisation formed by Apnalaya.	Have a reason to come out of the house
M5	21	Employed; Completed class 12	Hindu	Last-born with 1 sister at home. Lives with both parents.	Mother is member of self-help group (not very active).	Interest to work with girls in community
M6	21	Full time student; First year of university	Muslim	Second-born with 2 brothers and 1 younger sister. Lives with both parents.	Mentor recipient of educational scholarship from Apnalaya	Have a reason to come out of the house
M7	22	Computer teacher; First year of university (externally)	Muslim	First-born with 1 brother and 2 elder sisters. Lives with both parents.	Mentor recipient of scholarship	Additional income
M8	19	Full time student; Appeared for class 12 exams.	Hindu	Last-born with 2 brothers. Lives with both parents.	Mother is active member of self-help group	Learn and help girls in community
M9	21	Full time student; Appeared for class 12 exams.	Muslim	Last-born with 2 brothers and 1 sister. Lives with both parents.	Mother is active member; father participated in programs for men.	Learn and help girls in community
M10	22	Not employed; First year of university (externally)	Muslim	Last-born, lives with 1 brother, 1 sister and 1 nephew, and mother (father died recently)	Parents sought help for married sister’s domestic abuse.	Forming own identity/having recognition
M11	22	Not employed; Full-time mother. Completed class 12	Muslim	Lives with husband and in-laws.	Mentor recipient of scholarship	Learn and help girls in community
M12	19	Not employed; Completed class 12	Muslim	Second-born with 1 brother, his wife and 2 sisters. Lives with both parents.	Elder sister beneficiary of program for physically challenged.	Learn and have reason to come out of the house.
M13	18	Not employed; Completed class 12	Muslim	Third-born with 3 sisters. Lives with both parents.	Elder sister beneficiary of program for physically challenged.	Learn and help girls in community
M14	25	Employed; First year of university (externally)	Hindu	Third-born with 1 brother and his wife and 2 sisters. Lives with both parents.	Mother member of self-help group; all children recipients of scholarships	Learn and help girls in community
M15	18	Full time student; First year of university	Hindu	First-born with 1 brother. Lives with both parents.	Mother member of self-help group	Opportunity to learn

Mentors 11 to 15 dropped out before the implementation started and were not interviewed for the case studies. After the first day of the intensive training, M11’s family decided they could not deal with the logistics of caring for her small baby. The time commitment required to recruit athletes far exceeded what M14 and M15 could accommodate given their study and work commitments. Two sisters M12 and M13 had to withdraw as they went to live in their native village.

### Variations and changes in mobility restrictions

In Shivaji Nagar, parents restrict the movement of young women through ‘male’ public spaces, and their behaviour is closely policed by neighbours and relatives. Any deviation from expected patterns raises suspicion about relations with men or boys, and is interpreted by neighbours as a marker of bad parenting. The severity of mobility restrictions depends on the purpose of the outing, the time of day and the distance a girl travels. Going to school or work are considered ‘valid’ reasons, but leisure time with friends or acquaintances is not. At night when it is dark and during the heat of the afternoon when streets are relatively deserted, are times seen as especially unsafe. The immediate neighbourhood (a few houses in her lane) is considered safer than places further away like markets and public transport, which are associated with men and boys. Measures to protect a woman’s safety in public include sending an escort with her, mostly an older relative, though a younger brother is also acceptable.

**Fig. 1:**
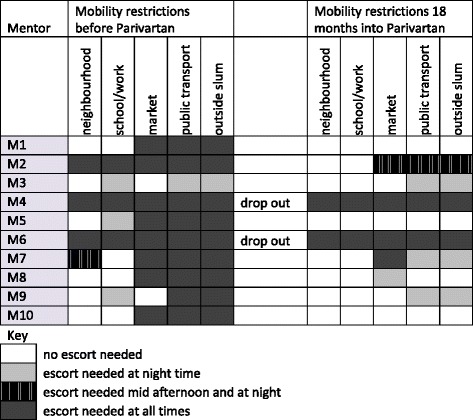
Variations and changes in mobility restrictions

The extent of mobility restrictions varied among mentors before and during the programme. We summarize variation in restrictions for moving to various places, by depicting whether she needed an escort at all times, during unsafe times in afternoon and evening, only after dark or not at all (Fig. 1). The graphic below visualises the variation in mobility restrictions before respondents began as mentors, and 18 months later. It demonstrates clearly that mentors were able to negotiate permission to move more freely on their own, both to pursue programme-related activities and beyond. Despite progress, limitations remained. Mobility restrictions were re-imposed upon those mentors who dropped out of the programme during implementation.

Various demands came with the role of mentor, with the need to move around in the community the most prominent one. We analyse the programme’s expectations and the challenges mentors encountered at sequential stages of training and implementation.

#### Initial residential gender training

At the start of the programme a 5-day residential training on gender equality, life skills and rights was held in a location outside the community. Staying away from home overnight predictably raised suspicion as expressed by one of the mentors:

“I was not sure if I would be allowed to be out for this training as it was for 5 days and we had to stay there. You know the people here talk so much about girls. If a girl is not seen in the house for one night they come asking, ‘Where is your daughter? She is not seen.’ They have nothing to do with it yet they will come and ask the mother or at least gossip with other women in the area. This is bad…. with this fear of bad name parents don’t send their daughter out in the night to stay somewhere else.” (M10, RND 01).

This strong community policing of women’s movement made negotiating mentors’ attendance difficult. Nevertheless, for nine of the 15 young women invited to the training, Apnalaya’s credibility as a respected grass-roots organisation engendered sufficient trust. For the other six mentors (M2, M5, M6, M11, M12 and M13) Apnalaya staff were involved in several levels of negotiation to persuade parents to let their daughter participate. M6’s mother was supportive but insisted that Apnalaya staff sought permission from the father and the elder brother. This took multiple home visits up to the last day before the training when the elder brother was available. A long conversation was interrupted by the arrival of a visitor, at which point the brother suddenly agreed and signed the consent form. Later that evening he forbade his sister to go as he had only agreed to avoid a discussion in front of visitors. However, M6 quietly left the house early in the morning while others were asleep, letting only her mother know. This resulted in an aggressive response from the father and elder brother banning the mother and younger brother from the home for three days. Upon her return home after the training, they did not speak to her for several days, yet M6 moved on to the next step in the programme: recruitment of young girls.

Spending 5 days and 4 nights together in a non-restrictive environment provided a safe space for the young women to discuss and question mobility and visibility restrictions imposed on them, while practicing negotiation skills to push boundaries. Another major outcome of the residential training was the building of strong personal bonds between the mentors, a powerful resource of social support for the recruitment and implementation stages of the programme.

#### Recruitment process of young girls into the Parivartan programme

A lot of the narratives on how mentors managed to push boundaries refer back to this period. The recruitment of athletes involved obtaining consent from families, a process that took much longer than anticipated. Mentors had to visit the families of the girls’ multiple times, initially accompanied by Apnalaya staff but later by themselves. Apart from contravening prevailing mobility norms, these visits encroached on time spent on household chores - both required negotiation at home. While some parents asked their daughter to quit the programme as time commitments far exceeded what had been communicated to them, the mentors stood their ground. As mentors felt safer to move through the community in little groups, they coordinated visiting the families together. Eventually, the inconvenience of organising times resulted in them going on their own, sometimes unbeknownst to the parents. Losing the safety of the group did feel uncomfortable and gave cause for anxiety. They feared both harassment by men and boys, and being seen by acquaintances and neighbours in areas where they are not expected to come on their own. All 10 mentors reported being confronted with these issues, but all came through adopting their own strategies. To avoid harassment by men, mentors approached their destinations through different roads or by-lanes when alone.

“Actually, the first time I went to XX without another mentor, I was also very scared. The entire area XX you will only see men around. And to go through this crowd initially was a big challenge we had. So we would get out through the internal lanes because we did not want to go through them. This is how it worked for us for 2-3 months but now we can easily tell the men to move and we make our way through the crowd even when alone.” **(M10, RND 01).**

Several mentors reflected on the constant negotiation with parents to counteract the effect of neighbours commenting on their movements through the community. Trust developed gradually through communication as the parents came to understand that the role as mentor demanded this level of mobility. Neighbours remained keen to police her movements, requiring further negotiations during the actual implementation of Parivartan.

#### Sports and reflective sessions with the athletes

As the actual programme started, mentors accompanied girls from their homes and dropped them back after each session. Sessions ran in the afternoon when streets and alleys are relatively deserted, and her visible presence lead parents, neighbours and relatives to request timings that seemed more appropriate or the company of an escort. Instead of compromising, mentors took it as a challenge to overcome these barriers. The fearless attitude displayed by some mentors (M 1, M3, M7 and M9) catalysed others to resist interference too. Many mentors acknowledged the power of sharing both the challenges and solutions from fellow mentors, leading to increased confidence as a group and as individuals.

Framing the greater mobility and visible presence in the community as a ‘duty’ of being a mentor and a requisite for doing ‘good work’ was the main strategy used in negotiating with parents and in silencing neighbours.

“Whenever I am late at the sessions or the meetings that Apnalaya has for us in this programme, the women around my house come and ask my mother, ‘where is she? It is so late? When will she come? She should not be out of the house for such long hours.’ That is when my mother tells them straight in their face, ‘she is out doing some good work and I am proud of my daughter. We (parents) trust her.’ When my mother says this they keep quiet for a moment, but the next time I am late they come and ask again, but my mother stands by me. I am happy that she understands my role as a mentor.” **(M5, RND 01).**

Reaching the school grounds where the sports sessions were held required a 45 min walk through markets and passing bus stops—all places crowded by men and boys. Mentors again coordinated with fellow mentors to travel together to accompany groups of athletes. While they reported incidents of teasing, name-calling or stalking, mentors and their athletes developed the ability to handle the harassment with increasing confidence.

Mentors perceived their increased confidence as the main personal benefit from their association with Parivartan and this boosted their commitment to the programme. As they examined the previously imposed restrictions on their mobility, they felt convinced that change was desirable and justified. From this position, it was easier to negotiate parents’ support to fulfil their mentor responsibilities.

#### Further training programmes outside the community

Eight months into the intervention, the programme needed the mentors to attend three non-residential trainings, each lasting 2 days and one 2-day residential training conducted outside the community. This required them to travel independently using public transport and coming home late in the evening. For mentors M1, M4, M5 and M6 this was the first time they travelled out of the community without a family member accompanying them. Yet the company of other mentors provided them with confidence and readiness to handle any harassment they might encounter.

### Hopes, strategies

Parents have clearly been pivotal in supporting their daughter to take on the role of mentor. Mentors attributed their parents’ support to the way they had strategically shared some of the content on gender and rights included in the reflection sessions for athletes:

“I discuss the topics that are in the module with my mother… but not everything together. I give her small small doses. This will help her understand what I am working towards and also know where my thinking is coming from. I have to see her mood and then go ahead talking to her… if I talk everything to her at the same time and it becomes an overdose then she can ask me to shut up.” **(M5, RND 02).**

The process of building trust was clearly a gradual one, navigated with care and predominantly via the mother. As social customs restrict direct interaction between young women and older men in the household, it was the mother who negotiated with her husband and sons. Yet five mentors (M1, M3, M4, M5 and M9) did report changes in direct communication with their father and all with their elder brothers to negotiate pushing boundaries. Several mentors narrated their father rebuffing neighbours, and justifying her participation in the programme and movement through the community.

“… like if I would go out he (father) would be worried and keep calling me. But now he knows that I know what to do when, so he is not much worried. Even if someone asks him what I am doing out for so long, he answers them appropriately” **[M5, RND 02].**

It seems that demonstrating her communication and negotiation skills within the family, and sharing her achievements as a mentor, has given her parents confidence in her ability to deal with situations outside. This opening for dialogue, with the trust it inspired, gave mentors the capability to negotiate mobility beyond her Parivartan duties, to pursue education but also for leisure time. Prior to Parivartan, mobility restrictions had caused three mentors (M1, M5 and M10) to drop out of mainstream education. Yet, with increased confidence and parental trust, M1 and M5 began attending night school outside the community; this required them to access public transport and travel back home in the dark, on their own.

Mentors M8 and M9 negotiated leisure time with their friends going beyond the slum boundary, taking pride in commuting independently, something inconceivable before the programme. They navigated this change partly by first obtaining permission to spend leisure time with the mentor group, an intermediary step that made it easier to gain permission from parents.

### Fears and failures

Mentors recounted their agency in effectively enrolling parents’ support for their duties as mentors and beyond, yet there was hesitation too. They did not take their current freedoms for granted and fears of ‘falling back’ were prevalent, with mobility seen as conditional on not making mistakes. Sharing their opinion with parents, demanding justice or asking to be involved in any decision that concerns her, was still seen as a potential ‘step too far’.

“I am scared that if I say something or ask for some permission, she [mother] could say “no there is no need for you to go out of the house anymore.” I fear that she will stop me from doing this as well. She will say, “you don’t go out at all,” and I will have to be in the house all the more. And that day she got angry with me and said, “don’t go to school and don’t go anywhere… just don’t get out of the house. There is no need for you to go out.” She was saying this.” **(M7, RND 02).**

Once mentors had started moving independently, they became frustrated when they needed a ‘valid’ reason to go out, or when only permitted out if escorted by a male relative. Ready and willing to negotiate, they clearly needed to be cautious and strategic in pushing boundaries gradually. Even when mentors felt they could appropriately respond to neighbours who might question their mobility, they still feared neighbours’ power to influence their parents. ‘Suspicion’ about sexual chastity and its impact on family honour could easily raise its head and undo the hard-won trust they had built. There was also concern about incidents in the community re-awakening doubts about how unsafe the environment really is, and how others may incite parents to re-instate normative restrictions.

Mentor 2, an orphan who lived with her grandmother saw her freedom reigned in when her uncle, who is her guardian, returned from the Middle East where he had been exposed to more orthodox religious ideas. Having described her relative freedom in the first round of interview, in the second she describes how his views had changed:

“….. he is come for the past one month and now everything in the house has changed. I have to be careful about the time I spend out of the house, the cloths I wear… he is insisting on every woman and girl in the house wearing a *burkha*. **…** He was not like this before, when grandmother spoke to him about me joining the programme he readily agreed and never had a problem with my clothing. But now since he is back, he is behaving as if he has lost his mind.”**(M2, RND 02).**

For M2, increased restrictions on clothing and the amount of time spent outside the house had become reality.

During the first round of interviews all mentors had spoken of their wish and resolve to postpone marriage until after the end of the Parivartan programme. By the second round, 6 months later, two mentors had dropped out because of marriage (M6) or engagement (M4). The decision to quit had been imposed by the families they were getting married into. Before their engagement they achieved freedoms equal to those of other mentors. As the group of mentors had become a new important reference group, they were acutely aware of what they had come to see as a ‘right’ now being revoked. The failure of their attempts to negotiate, which had been effective with parents previously, left them clearly exasperated:

“(T)he rules they have, I am unable to follow them and also to understand why they are saying this… They often say, ‘That’s why I say girls should not go out so much…. when they get outside air then they don’t like to stay at home’.” **(M6, RND 02).**

Her personal history is important to consider both during and before the programme. Mentor 6 was the one who disobeyed her elder brother about attending the residential training. Initially she and her parents had faced a lot of questions from neighbours about her movements. Yet everyone had become more supportive of her duties serving younger girls in Parivartan. This ultimately brought more respect to Mentor 6 and her family, with neighbours now approaching her for advice on education and schooling. In the first round of interview, Mentor 6’s level of agency was impressive. The start of marriage negotiations had actually predated Parivartan. She had already broken off one engagement as it meant moving back to her village which she refused. She narrated how she had successfully negotiated with her new fiancé to postpone her marriage until after Parivartan as he lived about 20 km away, which would preclude her continuation. Unfortunately, her fiancé’s mother became ill and insisted that her son get married at the earliest. This was a shock to the mentor, and again she negotiated with her fiancé to travel home every weekend in order to continue her association with the programme. But once she got married and went to live with her in-laws, she was not allowed out, and she was required to wear a *burkha* even in front of her male in-laws. Her husband assured her that everything would be fine later but patience was called for in these early days of marriage as opposing his mother’s wishes would send the wrong message. She never did get permission to go back home on weekends and thus quit as a mentor. She reported enjoying the times she had on her own with her husband, trusting him and understanding the pressure he is under from his family.

For mentor 4, the renewed restrictions were imposed in her own home. Her story illustrates how her parents seemed ‘pressured’ to do a U-turn and start conforming back to normative expectations of confining women to household chores, and away from public visibility. M4 described the changes in her mother and fiancé’s behavior towards her, after her future mother-in-law had come to stay with her family, and had been unimpressed with the freedom she was afforded. Her fiancé started exerting control on her while she was still leading *kabaddi* sessions as a mentor, checking her whereabouts by phoning both her and her mother.

“On Sundays I used to go late, roam around and then go to the market, but he (fiancé) would call me often and start yelling at me. “How much time are you taking? So much time is being spent at work. In half an hour you should be at home and you are roaming around till late. And mother is working alone at home.” Then they coax my mother too. So when I would return home, my mother would also yell at me “What is it about you, go early, come back early.” Otherwise I will not let you go. If you are going, then come back early.” **(M4, RND 02).**

Mentor 4 had perceived her father struggling with the apparent demands and attitudes of her future in-laws. She observed him expressing doubts to his wife, who rebuked him with “everything is new, let it go now and see what happens later”. Her father had become quiet and her mother seemingly tried to create a conducive environment in preparation for marriage. Again, personal history seems important in illuminating her parents’ conflicted situation as they had concerns about her mental fragility and health impacting on her marriageability. She was once kissed by a male childhood friend, which upset her to the extent she took pills to end her life. The risk of breaking off an engagement and the gossip it would create in the community, further decreases the prospects of marriage. Receiving no support and only silence from her parents, the mentor finally gave into the demands of the in-laws and her fiancé to discontinue with the programme. Once she quit, she was confined to the house and lost all contact with the other mentors.

## Discussion

This qualitative case study situated in a Mumbai slum demonstrates how a carefully structured sports mentoring programme, conceptualized to contest restrictive visibility norms for girls and women in public spaces, allowed mentors to effectively negotiate a ‘respectable’ presence outside the home for themselves and the participating girls. The Parivartan programme relied heavily on the mobility and visibility of mentors for its success and charted out a comprehensive strategy that supported their collective and individual agency and promoted proactive family engagement. Not compromising on the women’s ability to negotiate their own movements and reputation (while avoiding reproducing paternalistic norms of male protection and escorting) proved instrumental in the ‘transformative’ nature of this intervention. The mentors themselves negotiated freedoms both in and outside their homes. They changed the nature of key day-to-day social interactions, whether it was with mothers, fathers and brothers, neighbours, parents of their groups of athletes, or men on the streets. We must reiterate that mentors were purposively selected as ‘positive deviants’, educated and still unmarried in their early 20s, from families previously involved in development initiatives or self-help groups with Apnalaya, a respected organisation in the community. Since social norms set limits to bargaining, and to what can be bargained over [15], opportunities for shifts in gender relations were maximized by involving families supportive of their daughters’ aspirations. Our data suggests that elevated levels of aspirations on the part of mentors and their mothers was a strong driver of the bargaining in favour of mobility within and beyond the family.

Contesting restricted norms involved gradual negotiation, initially drawing on ‘valid reasons’ to gain permission to perform necessary tasks or ‘duties’ as mentors. Careful bargaining was needed to navigate the complex shift from an identity constructed around predominately domestic duties towards a new identity of a mobile young woman working towards a cause, visible to neighbours and community. The bargaining power to change gender dynamics at home and in the community are linked [15]. The most critical enablers at home were the mothers with whom mentors shared discussion topics on gender inequality and gender justice, always strategic about what and when to share. This process was characterised by both cooperation and conflict; the more mentors questioned and challenged issues of inequality, the less they were willing to accept constraints on their mobility and freedom. In line with traditional gender conduct, mothers initially negotiated on their behalf with the male members of the family, yet mentors also started communicating directly with elder brothers and fathers. This demonstration of individual agency in itself seems to have contributed to the mentor winning parental trust in her ability to be safe in public spaces. The family was thus enrolled in the co-production of a new identity [16] as a respectable young woman doing ‘good work’ with younger members of the community. Parents were active players by not giving into social sanctions of neighbours and relatives.

As the programme unfolded, the mentors developed into a strong support group for each other, influencing and inspiring each other to stretch boundaries, gaining confidence each time they witnessed each other’s successes. The programme created opportunities for collective action, increasing mentors’ ability to think and relate in a collectivized manner. They formed a new reference group for each other in terms of what was possible and acceptable. Our data corroborate the finding that collective bargaining and action are central to both questioning and contesting gender relations [15]. Mentors contested mobility restrictions taking risks as a group, with collective agency an important step towards greater individual agency in day-to-day social interactions [11]. First, they sensibly used strategies for protection: moving in groups, taking inside alleys to avoid crowds of men on the main streets when going on their own. In other words, they modified their behaviour to avoid unwanted male attention in order to resist exclusion from public space. While this strategy risked reproducing the norms that reduce their claim to safe access [17], the skills gained from the gender training enabled them to go further. Mentors rebuked men and boys on the street and came to no harm. They managed, gradually, to achieve the right to ‘take risks’ and put their right to access public spaces above their desire for protection, diminishing the perpetuation of patriarchal power [6]. Most mentors went beyond negotiating mobility for performing duties and achieved more leisure time away from home.

Notable in our data was how conversations steered away from reputational risks. The discourse of sexual safety was side stepped in both the narratives and in the intervention. In fact, the mentors’ presence in public spaces became de-sexualised. Instead of losing respectability by being visible in the community, they gained social standing through their new identity as mentor, performing a valued community service in association with a reputable and trusted social development organisation. This observation may have important implications for other interventions that aim to increase education and delay marriage. By dwelling too much on marriageability, programmes may unwittingly reinforce concerns about sexual safety rather than defusing them.

All mentors expressed anxiety about potential curtailment of their freedom, and insecurity about what will happen once the programme ends. Importantly, bargaining power to leave the home all but evaporated for the two mentors who dropped out. Confronted with more gender-retrogressive in-laws, their parents’ support also waned. While they seemed to come from less progressive families in the first place, we cannot tell how the other mentors will fare once the marriage process starts. Mobility restrictions did increase again for the mentor whose uncle and guardian migrated back from the Middle East. Identity and ‘cultural’ politics appeared to take center stage at the cost of women and girls symbolically representing cultural exclusiveness.

Limitations to this study include a potential desirability bias in mentors’ accounts, even when they felt free to express concerns about their future. We also cannot know how the mentors’ income increased their bargaining power within the family and what will happen when it falls away, but we will explore this once the third round of data, collected a year after the end of the programme, are available. We cannot disregard influences from secular change and other development initiatives. Indeed, previous efforts addressing gender inequality no doubt contributed to the mentor families’ readiness for change, in the same way this sports programme inspires and prepares other families for future change opportunities. The ultimate target of Parivartan are the girls participating in the sessions, and documenting the changes among them was beyond the scope of this paper. However, a first step was establishing that mentors drawn from within the slum community were able to implement the programme and provide new role models.

Despite visible change in these young women’s presence in public spaces, it seems too early to claim that these mentors have set a new standard by having adopted new roles and responsibilities [12]. Yet the symbolic aspects of performing gender came under strong scrutiny and the positive reception so far gives hope for greater participation of young women as citizens in times of ongoing social and economic change.

## Conclusion

In a Mumbai slum where fear and social control significantly limit young women’s access to public space, the day-to-day social interactions of mentors implementing a sports-based programme changed in significant ways. The mentor role created opportunities for collective action, increasing mentors’ ability to think and relate in a collectivized manner and taking risks free from overbearing paternalistic protection. This paper makes a significant contribution because gender norms continue to act as structural barriers to many public health programs and rob generations of young women to participate as citizens in society. It provides important insights into addressing embedded social norms that perpetuate gender discriminatory practices and the social patterning of health inequalities.

## Abbreviations

ICRW: International Center for Research on Women
IIPS: International Institute of Population Sciences
NGO: Non-Government Organisation
